# Sulfate Minerals: A Problem for the Detection of Organic Compounds on Mars?

**DOI:** 10.1089/ast.2014.1160

**Published:** 2015-03-01

**Authors:** James M.T. Lewis, Jonathan S. Watson, Jens Najorka, Duy Luong, Mark A. Sephton

**Affiliations:** ^1^ Impacts and Astromaterials Research Centre, Department of Earth Science and Engineering, Imperial College London, London, United Kingdom.; ^2^ Impacts and Astromaterials Research Centre, Department of Mineralogy, Natural History Museum, London, United Kingdom.

## Abstract

The search for *in situ* organic matter on Mars involves encounters with minerals and requires an understanding of their influence on lander and rover experiments. Inorganic host materials can be helpful by aiding the preservation of organic compounds or unhelpful by causing the destruction of organic matter during thermal extraction steps. Perchlorates are recognized as confounding minerals for thermal degradation studies. On heating, perchlorates can decompose to produce oxygen, which then oxidizes organic matter. Other common minerals on Mars, such as sulfates, may also produce oxygen upon thermal decay, presenting an additional complication. Different sulfate species decompose within a large range of temperatures. We performed a series of experiments on a sample containing the ferric sulfate jarosite. The sulfate ions within jarosite break down from 500°C. Carbon dioxide detected during heating of the sample was attributed to oxidation of organic matter. A laboratory standard of ferric sulfate hydrate released sulfur dioxide from 550°C, and an oxygen peak was detected in the products. Calcium sulfate did not decompose below 1000°C. Oxygen released from sulfate minerals may have already affected organic compound detection during *in situ* thermal experiments on Mars missions. A combination of preliminary mineralogical analyses and suitably selected pyrolysis temperatures may increase future success in the search for past or present life on Mars. Key Words: Mars—Life detection—Geochemistry—Organic matter—Jarosite. Astrobiology 15, 247–258.

## 1. Introduction

Organic matter detection missions sent to Mars, the Viking landers and Mars Science Laboratory (MSL), have so far offered no conclusive evidence of organic matter preserved on the planet's surface. The failure to detect organic matter *in situ* is paradoxical when it is considered that comets, meteorites, and interplanetary dust particles have been contributing organic compounds to the Red Planet throughout its history (Atreya *et al.,* 2007). In addition, organic carbon phases have been detected in martian meteorites (Sephton *et al.,* 2002; Steele *et al.,* 2012) and martian volcanoes; life and serpentinization could also have produced organic compounds in the past and may still do so today (Atreya *et al.,* 2007). Organic matter is therefore either accumulating at levels below detection limits, being destroyed by martian processes (Benner *et al.,* 2000; ten Kate *et al.,* 2005), or its presence is obscured through mission sample processing and analytical procedures.

Thermal extraction is a common *in situ* analysis approach (Mahaffy, 2008). However, minerals are known to influence the composition of thermal extracts (Saiz-Jiminez, 1994; Faure *et al.,* 2006). For example, any mineral species that decomposes to give oxygen on heating may oxidize organic matter present in the sample of interest. A mineral class that has received a great deal of recent attention is the perchlorates, which are salts containing chlorine and oxygen that thermally decompose to give molecular oxygen on heating (Devlin and Herley, 1986). The Phoenix lander detected perchlorates in northern martian soils with the Microscopy, Electrochemistry, and Conductivity Analyzer (MECA) instrument (Chevrier *et al.,* 2009; Hecht *et al.,* 2009; Kounaves *et al.,* 2010, 2014b). Perchlorate species predominantly decompose at ≤600°C (Shimokawabe *et al.,* 1977), so their presence on Mars is further implied by Phoenix Thermal Evolved Gas Analyzer (TEGA) results, which show the evolution of oxygen in the 300–600°C range (Hecht *et al.,* 2009). A corresponding release of carbon dioxide detected between 200°C and 600°C was tentatively interpreted as resulting from the combustion of organic matter facilitated by perchlorate-sourced oxygen, though no intact organic matter was detected (Ming *et al.,* 2009). Similar interpretations have recently been applied to historical Viking pyrolysis data (Navarro-González *et al.,* 2010). Perchlorates may form on Mars by gas phase oxidation and are probably widespread in the arid martian soils (Catling *et al.,* 2010). In addition, perchlorate brines may be stable for brief periods of time at the Phoenix landing site (Chevrier *et al.,* 2009). Perchlorates are a possible cause of the detection of chlorinated hydrocarbons and oxygen by the Sample Analysis at Mars (SAM) instrument on MSL (Glavin *et al.,* 2013). The martian meteorite EETA79001 was found to contain 0.6±0.1 ppm perchlorate (Kounaves *et al.,* 2014a).

A relatively neglected class of mineral that can decompose to produce atomic oxygen during heating is the sulfates (Holt and Engelkemeir, 1970). The sulfate ion decomposes to sulfur trioxide and then, at higher temperatures, sulfur dioxide and atomic oxygen (Bailey and Smith, 1972). Sulfur trioxide can also combine with water to form sulfuric acid (Wong *et al.,* 2003). The presence of sulfates in mixtures introduced to pyrolysis chambers could therefore pose a similar problem to that observed for perchlorates. If the sulfate is hydrated, then the potential generation of sulfuric acid is an additional complication. Thermogravimetric studies show that most sulfates decompose at temperatures greater than 600°C (Table 1); however, iron and aluminum sulfates begin to decompose at temperatures similar to those used for the analysis of macromolecular organic material in pyrolysis studies (Mu and Perlmutter, 1981; Sephton and Gilmour, 2001; Sephton *et al.,* 2004, 2013; Navarro-González *et al.,* 2006; Sephton, 2012).

**Table 1.** Temperature at Which the Sulfate Ion Begins to Decompose to Give Sulfur Dioxide during Thermal Decomposition of Different Sulfate Species

*Mineral*	*Formula*	T *at which sulfate decomposition begins (°C)*	*Decomposition atmosphere*
Jarosite	KFe_3_(OH)_6_(SO_4_)_2_	501^a^	Nitrogen
Natrojarosite	NaFe_3_(OH)_6_(SO_4_)_2_	555^a^	Nitrogen
Hydronium jarosite	H_3_OFe_3_(OH)_6_(SO_4_)_2_	557^b^	Nitrogen
Ammonium jarosite	NH_4_Fe_3_(OH)_6_(SO_4_)_2_	510^c^	Nitrogen
Plumbojarosite	Pb_0.5_Fe_3_(OH)_6_(SO_4_)_2_	531^a^	Nitrogen
Argentojarosite	AgFe_3_(OH)_6_(SO_4_)_2_	548^d^	Nitrogen
Alunite	KAl_3_(OH)_6_(SO_4_)_2_	610^e^	Air
Natroalunite	NaAl_3_(OH)_6_(SO_4_)_2_	590^e^	Air
Hydronium alunite	H_3_OAl_3_(OH)_6_(SO_4_)_2_	680^e^	Air
Ammonium alunite	NH_4_Al_3_(OH)_6_(SO_4_)_2_	660^e^	Air
Ferric sulfate	Fe_2_(SO_4_)_3_	494^f^	Nitrogen
Aluminum sulfate	Al_2_(SO_4_)_3_	580^f^	Nitrogen
Lead sulfate	PbSO_4_	759^a^	Nitrogen
Magnesium sulfate	MgSO_4_	780^f^	Nitrogen
Sodium sulfate	Na_2_SO_4_	1100^g^	Nitrogen
Calcium sulfate	CaSO_4_	1200^h^	Nitrogen

^a^ Frost *et al.* (2005a).

^b^ Frost *et al.* (2006a).

^c^ Frost *et al.* (2006b).

^d^ Frost *et al.* (2005b).

^e^ Rudolph *et al.* (2003).

^f^ Mu and Perlmutter (1981).

^g^ Samadhi *et al.* (2001).

^h^ West and Sutton (1953).

Jarosite minerals are members of the alunite supergroup [minerals with the general formula AB_3_(TO_4_)_2_(OH)_6_]. In jarosite, the general formula is characterized by having Fe^3+^ in the B site and sulfur occupying the T site (Basciano and Peterson, 2008). On Earth, a natural solid solution exists in the A site of potassium, sodium, and hydronium ions (Brophy and Sheridan, 1965). The A site can also be occupied by ammonium, lead, or silver ions (Drouet and Navrotsky, 2003). It is well known that variation of the cation species in the A and B sites influences the decomposition temperature of the jarosite structure and that other sulfate species also have highly variable decomposition temperatures. Hydronium jarosite will undergo complete decomposition from 619°C when intermediate ferric sulfate breaks down to give iron oxide and sulfur dioxide [sulfuric acid is also produced as a decomposition intermediate and breaks down to give sulfur dioxide at 557°C (Table 1)] (Frost *et al.,* 2006a). Sulfur trioxide is an intermediate in sulfate decomposition, and it breaks down to give atomic oxygen and further sulfur dioxide (Bailey and Smith, 1972). With other forms of jarosite, the iron in the B site of the general formula will still form a ferric sulfate intermediate, which decomposes at temperatures that are problematic for organic molecule detection, but the A site cation will form a more stable sulfate intermediate, which does not decompose until higher temperatures (Frost *et al.,* 2005a). Additional metal oxides, that are unrelated to the breakdown of sulfate intermediates, form during jarosite decomposition; the oxygen is sourced from the hydroxyl groups within jarosite, so these metal oxides do not act as a sink for oxygen released during the breakdown of sulfate ions within jarosite (Frost *et al.,* 2005a).

Jarosite is a common mineral in acidic, sulfur-rich environments (Baron and Palmer, 1996). Sulfates, including jarosite, are common on Mars and are interpreted as evidence of surface and near-surface waters present at the time of their formation (Ehlmann *et al.,* 2009). Jarosite can form by the aqueous oxidation of pyrite deposits in a saline, low pH, low water-to-rock-ratio environment (Zolotov and Shock, 2005). Such an environment is thought to have existed in the late Noachian and Hesperian when outgassing from volcanoes released abundant sulfur and water into the martian atmosphere (Bibring *et al.,* 2006). Noachian and Hesperian terrains are well preserved and widespread on Mars (Michalski *et al.,* 2013). Jarosite is preserved for geological timescales only in extremely arid conditions, and the persistence of jarosite on Mars is testament to the planet's extreme surface aridity in post-Hesperian times (Elwood Madden *et al.,* 2004).

Sulfates are not just restricted to ancient terrains; the well-mixed dust that covers most surfaces on Mars has high concentrations of sulfates, at an average concentration of 5.82 wt % (Gellert *et al.,* 2004). Magnesium sulfate is a common cementing agent in the upper few centimeters of martian soil, making up approximately 10 wt % (Vaniman *et al.,* 2004). *In situ* analyses by landers and rovers support the assertion of widespread sulfates on Mars. Sulfur was detected in martian soils by the Viking landers at concentrations of 8–15%, with magnesium and sodium sulfates being inferred as the most common mineral phases present (Clark and Van Hart, 1981). At the Pathfinder landing site, the Sojourner rover Alpha Proton X-ray Spectrometer (APXS) detected soils that contained a mean value of 6.79% sulfur (Wanke *et al.,* 2001). The Opportunity Rover identified the ferric sulfate species jarosite and possibly gypsum at its Meridiani Planum landing site (Klingelhöfer *et al.,* 2004), and jarosite was present in rocks of the Burns Formation at an abundance of 29% of iron species, the other species being hematite, olivine, pyroxene, and magnetite (Morris *et al.,* 2006). While studying Gusev Crater, the Spirit Rover became embedded in sand in which ferric sulfates were a major phase (Arvidson *et al.,* 2010). Sulfur dioxide peaks seen in MSL SAM results from the Rocknest site in Gale Crater were variable but evolved in the form of two main peaks between 450°C and 800°C, and were described as consistent with the decomposition of iron sulfates (Leshin *et al.,* 2013). However, analyses at both Rocknest and the Yellowknife Bay study site by the Chemistry and Mineralogy (CheMin) instrument of MSL found only calcium sulfates, suggesting that if iron sulfate is present it exists at levels below detection limits at these locations (Bish *et al.,* 2013; Vaniman *et al.,* 2014). Oxidized sulfur species are widespread in martian meteorites, with calcium and magnesium sulfates being the most common forms (Burgess *et al.,* 1989; Gooding, 1992). Mars analog sites on Earth, such as Río Tinto and Panoche Valley, are notably sulfate-rich (Navarro-González *et al.,* 2006).

In this paper, we examine sulfate minerals that are known to exist on Mars, focusing on jarosite, and assess their influence on organic matter detection by thermal extraction methods. The widespread and abundant nature of sulfates suggests that any effects will be almost unavoidable for *in situ* Mars analyses. We also compare the behavior of these sulfates with other common Mars-relevant mineral phases. Our data provide guidance for both interpreting existing data from Mars but also for directing the future operation of thermal extraction units by suggesting routes to mitigate any negative effects of sulfate decomposition on organic detection.

## 2. Materials and Methods

### 2.1. Samples

A number of Mars-relevant sulfates and other mineral types were obtained for this study. A natural jarosite clay was collected from Brownsea Island, Dorset, UK. On the southern coast of the island, subhorizontal beds of the Branksome Sand and Parkstone Clay are well exposed in the short cliffs. The Parkstone Clay unit is rich in lignite and contains pyrite. The iron sulfide oxidizes to jarosite on rock surfaces, which then further oxidizes to iron oxide at the base of the cliffs. Jarosite-enriched layers were removed from the cliff face by hammer or by hand.

A natural jarosite clay sample, rather than a pure synthetic standard, was used for this work for two reasons. Jarosite analyzed *in situ* on Mars will likely be present as a small percentage of a well-mixed sample (Morris *et al.,* 2006). Secondly, the jarosite structure can undergo multiple substitutions (Basciano and Peterson, 2008). As cations exert a significant influence on decomposition temperature (Table 1), a natural jarosite clay was chosen so that the decomposition was representative of a natural cation ratio rather than a synthetic sample, which may be unnaturally enriched in a particular cation. In preparation for analysis and the main experimental work, a whole rock sample of the jarosite clay was crushed in a Tema mill.

For comparison with, and deconvolution of, the signals in the natural jarosite clay, a number of individual minerals were obtained. Laboratory standards of ferric sulfate hydrate, calcium carbonate, goethite, and quartz were sourced from Sigma-Aldrich. Quartz also acted as the procedural blank. Gypsum was acquired from BDH reagents and chemicals. Siderite was synthesized in the laboratory. Illite (IMt-1), kaolinite (KGa-1b), and montmorillonite (Swy-2) were obtained from The Clay Minerals Society. As these are pure single-phase standards, they are not directly Mars relevant, but studying their decomposition augments our analysis of the natural jarosite clay decomposition and our interpretation of the relative behavior of different sulfate species on Mars.

### 2.2. X-ray diffraction (XRD)

The natural jarosite clay was analyzed by XRD. A subset of the specimen was ground and homogenized in a pestle and mortar and mounted in a flat holder within a Panalytical X'Pert Pro Alpha-1 system. The powder was analyzed between 5 and 90 °2*Θ* for three and a half hours under copper radiation with an X'Celerator detector. The diffraction pattern was analyzed with the X'Pert HighScore program with reference patterns from the ICDD PDF-2 database. The X'Pert software was used to perform a Rietveld refinement allowing quantification of crystalline phases in the sample to an accuracy of 1–3%.

### 2.3. Quantifying carbon in the natural jarosite clay

To assess the quantity and nature of carbon in the natural jarosite clay, a 3 g sample was washed in 1 *M* hydrochloric acid. The acid was first pipetted dripwise onto the natural jarosite clay, but no effervescence was observed. So the sample was mixed with 10 mL of the acid. A centrifuge was used to separate the acid from the solid, and it was pipetted off and replaced with fresh acid (×3). The natural jarosite clay was then rinsed in deionized water until the pH returned to 7. Samples of the untreated and acid-treated natural jarosite clay were analyzed by elemental analyzer–isotope ratio mass spectrometry (EA-IRMS) by Iso-Analytical Limited. EA-IRMS allows the total carbon in the sample to be measured. The difference in total carbon between the untreated and acid-treated samples allows the ratio of organic carbon to carbonate to be assessed.

### 2.4. Solvent extraction

To examine the extractable organic matter within the sample, 2 g of the natural jarosite clay was divided equally between two test tubes, and each sample was extracted with 5 mL of a 95:5 dichloromethane:methanol solvent mixture. The tubes were sonicated and then centrifuged, and the supernatant was pipetted off into a third test tube. The extraction was repeated three times, with the resulting supernatants added to the third test tube. The total supernatant was evaporated under nitrogen and then stored in a vial before subsequent analysis.

### 2.5. Pyrolysis–gas chromatography–mass spectrometry (Py-GC-MS)

Powdered samples were placed into quartz pyrolysis tubes and held in place by quartz wool. With all samples, other than siderite, 4 mg of sample was used; the siderite experiment used only 3 mg, as the response of carbon dioxide is much greater for siderite relative to the other minerals. The pyrolysis tubes were placed inside the platinum coil of a CDS 5200 pyroprobe under helium and heated at a rate of 20°C ms^−1^ to the target temperature, where it was held for 15 s. The interface was held at 150°C and coupled to a gas chromatograph–mass spectrometer for direct injection. For the natural jarosite clay and ferric sulfate hydrate experiments, individual samples (*i.e.*, samples run at one temperature step only, rather than using stepped pyrolysis) were run at 100°C intervals, the lowest at 400°C and the highest at 1000°C, allowing both the products of pyrolysis and the variation in decomposition rate at different temperatures to be investigated. An additional individual analysis at 550°C was used to focus on decomposition around typical pyrolysis temperatures used for studying macromolecular organic material (Mu and Perlmutter, 1981; Sephton and Gilmour, 2001; Sephton *et al.,* 2004, 2013; Navarro-González *et al.,* 2006; Sephton, 2012). For each of the other minerals being studied, two individual experiments were carried out, one at 600°C and one at 1000°C to show the decomposition characteristics at both typical pyrolysis temperatures and at the higher temperature to identify any species released under the full range of pyrolysis conditions. Additional individual natural jarosite clay samples were pyrolyzed at 600°C and 700°C with the gas chromatograph–mass spectrometer optimized for organic molecule detection (as described in the next section).

### 2.6. Gas chromatography–mass spectrometry (GC-MS)

The products of pyrolysis were analyzed by GC-MS with an Agilent Technologies 6890 gas chromatograph coupled to a 5973 mass spectrometer. The gas chromatograph injector was held at 200°C and operated in split mode (35:1) with a column flow rate of 2 mL min^−1^. Separation was performed on a J&W GS-Q PLOT column (30 m×0.32 mm). The gas chromatograph oven was held for 5 min at 35°C and then ramped at a rate of 10°C min^−1^ to 200°C, where it was held for 4 min. Mass spectra were acquired in the scan range 10–150 amu. In the natural jarosite clay and ferric sulfate hydrate experiments, the peak areas were normalized by sulfate mass in each run. We assumed 100% for the ferric sulfate hydrate standard and used the abundance of jarosite found by XRD for the natural jarosite clay.

To examine the products of natural jarosite clay pyrolysis for the presence of organic compounds, the gas chromatograph injector was held at 270°C and operated in split mode (65:1) with a column flow rate of 1.1 mL min^−1^. Separation was performed on a J&W DB-5MS UI column (28.9 m×0.25 mm×0.25 *μ*m). The gas chromatograph oven was held for 2 min at 35°C and then ramped to 300°C, where it was held for 8 min. Mass spectra were acquired in the scan range 45–550 amu.

For the analysis of the solvent extract of the natural jarosite clay, an Agilent Technologies 7890A gas chromatograph coupled to a 5975C mass spectrometer was used. The gas chromatograph injector was held at 200°C and operated in splitless mode with a column flow rate of 1.1 mL min^−1^ and a solvent delay of four and a half minutes. Separation was performed on a J&W DB-5MS UI column (30 m×0.25 mm×0.25 *μ*m). The gas chromatograph oven was held for 2 min at 40°C and then ramped to 310°C at a rate of 5°C min^−1^ to 310°C, where it was held for 14 min. Mass spectra were acquired in the scan range 40–550 amu.

## 3. Results

### 3.1. Phases present in the natural jarosite clay sample

Quantification by XRD showed that jarosite made up 5% of the natural jarosite clay sample from Brownsea Island. Jarosite is concentrated on rock surfaces, with the bulk of the sample made up of quartz (40%), goethite (36%), and clay minerals (kaolinite, 10%; illite, 9%). XRD pattern-matching indicates that the jarosite has a composition between the potassium and hydronium end-members.

### 3.2. Carbon in the natural jarosite clay

Both the untreated and acid-treated natural jarosite clay were found to have a carbon content of 0.45% by EA-IRMS, indicating that there is no significant carbonate component in the sample and that the carbon observed is organic carbon.

### 3.3. Organic compounds present in the natural jarosite clay sample

Solvent extraction indicated that the carbon in the natural jarosite clay contains extractable organic matter indicative of higher plant matter (undecane, heptadecane, octadecane, eicosane, methyl tetradecanoate, hexanol, hexadecanoic acid, octadecenoic acid, and octadecanoic acid). The natural jarosite clay was also contaminated by the plasticizer *N*-butyl-benzenesulfonamide.

### 3.4. Decomposition products of ferric sulfates

**FIG. 1.**
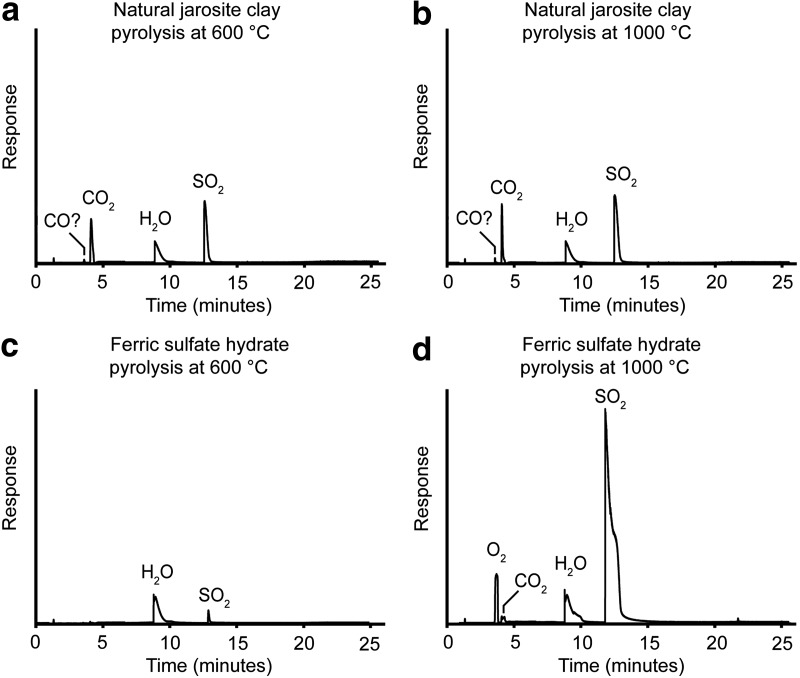
Total ion current chromatograms of the gaseous products released during individual heating experiments of samples of a natural jarosite clay and a lab standard of ferric sulfate hydrate at 600°C and 1000°C. The *m/z* 28 peak is labeled as CO?, as it could be either carbon monoxide or nitrogen; we infer carbon monoxide as discussed in the text. Very minor peaks for oxygen and carbon dioxide were present in the ferric sulfate hydrate experiment at 600°C but cannot be seen at the scale of the figure. All chromatograms are presented at the same scale.

**FIG. 2.**
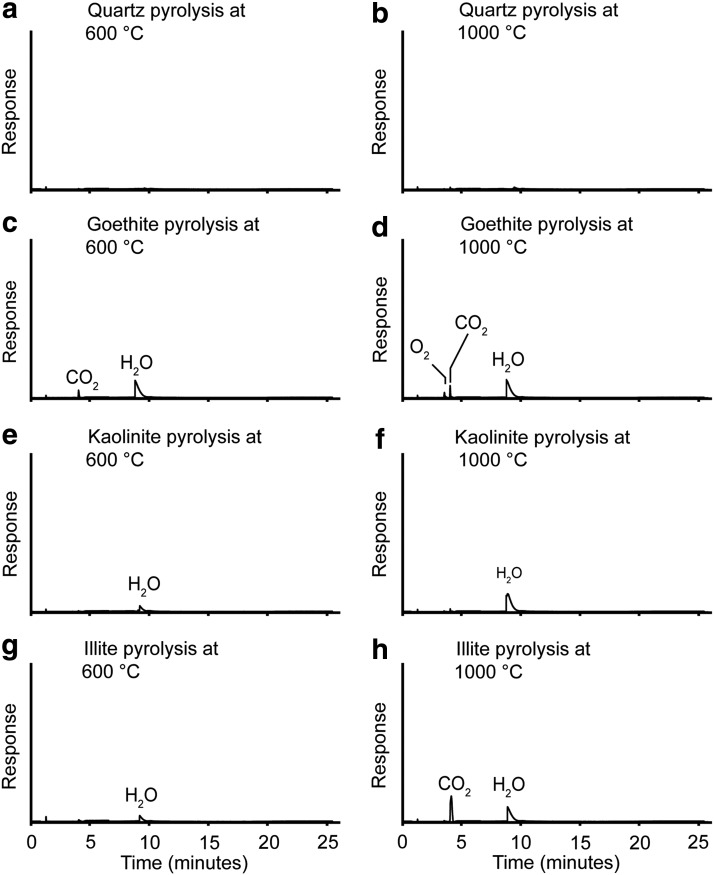
Total ion current chromatograms of the gaseous products during individual heating experiments of lab standards representing the non-sulfate mineralogy of the natural jarosite clay sample. All chromatograms are presented at the same scale.

**FIG. 3.**
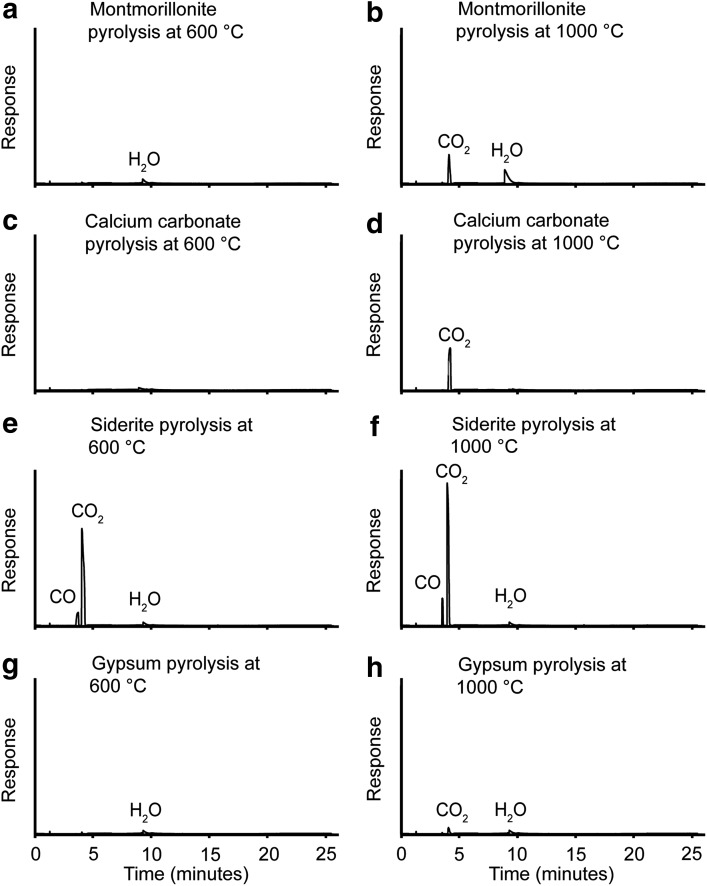
Total ion current chromatograms of the gaseous products during individual heating experiments of lab standards representing other Mars-relevant minerals at 600°C and 1000°C. All chromatograms are presented at the same scale.

The major gases released by the natural jarosite clay at 600°C and 1000°C were carbon dioxide, water, and sulfur dioxide (Fig. 1). A minor peak of mass-to-charge ratio (*m/z*) 28 was also detected at both temperatures, which could be either nitrogen or carbon monoxide, as both species have the same mass and retention time. We infer that this species is more likely to be carbon monoxide, as it is an expected product of partial combustion, the peak area increased with temperature, and no *m/z* 28 peak was seen in any other sample apart from siderite (Figs. 2 and 3). Oxygen was not detected at any temperature step for the natural jarosite clay.

The lack of carbonate phases in the XRD data for the natural jarosite clay, along with identical EA-IRMS total carbon values for the untreated and acid-treated samples, indicates that the carbon dioxide detected was due to the breakdown of organic matter and not carbonate decomposition. Pyrolysis data for untreated and acid-treated natural jarosite clay samples are almost identical, further indicating the absence of significant carbonate, because pyrolysis of a carbonate-containing sample would have generated much less carbon dioxide after being treated with acid. The substantial peak for sulfur dioxide indicates decomposition of the sulfate ions within jarosite and therefore decomposition to sulfur trioxide and then sulfur dioxide and oxygen (Bailey and Smith, 1972). However, as neither sulfur trioxide nor oxygen were detected directly, we infer that sulfur trioxide broke down rapidly and oxygen was completely consumed in the oxidation of organic matter. The oxidation of organic matter is therefore likely responsible for the majority of the carbon dioxide detected. Py-GC-MS of the natural jarosite clay, scanning in the range 45–550 amu with a DB-5MS UI column, found only that sulfur dioxide was detectable in the pyrolysis products, with a very minor benzene peak at 700°C. None of the organic compounds detected through solvent extraction were detected through Py-GC-MS. At pyrolysis temperatures, these compounds were converted to carbon dioxide. Our observations suggest that jarosite decomposition can act as an oxidizer of organic matter.

At 600°C, the lab standard of ferric sulfate hydrate produced distinct peaks for water and sulfur dioxide and extremely minor peaks for carbon dioxide and oxygen (Fig. 1). The sulfur dioxide peak area was substantially greater at 1000°C and was accompanied by an oxygen peak. Carbon dioxide was present at low levels during both the 600°C and 1000°C temperature steps. Only in the 1000°C experiment was the response great enough to be clearly visible in the chromatogram (Fig. 1). The carbon dioxide data suggest the thermal decomposition of a mineral impurity or minor contamination by organic compounds in the lab standard. Decomposition of sulfate ions can produce detectable oxygen during Py-GC-MS. Sulfur trioxide was not detected, suggesting it breaks down rapidly to sulfur dioxide and oxygen at temperatures equal to or greater than 600°C.

### 3.5. Decomposition products of other mineral phases

The clay minerals and goethite present in the natural jarosite clay could be responsible for a major proportion of the water evolved during heating (Fig. 2). For the goethite standard, oxygen was present at low levels during both the 600°C and 1000°C experiments. However, only at 1000°C was the response clearly visible at the scale of the chromatogram. To investigate whether goethite could have contributed significant oxygen during decomposition of the natural jarosite clay, pyrolysis of the goethite standard was repeated with individual experiments at 100°C increments between 600°C and 1000°C. When plotted on the same axes as the natural jarosite clay data, oxygen released by the goethite standard plots as a flat line and can be ruled out as a significant contributor of oxygen in the natural jarosite clay heating experiment. A minor carbon dioxide peak was seen at both temperatures, which suggests the presence of minor organic contamination in the lab standard. A distinct carbon dioxide peak was seen at 1000°C for illite, but none was detected at 600°C. The data suggest decomposition of a carbonate impurity, as organic matter would have suffered substantial decomposition at 600°C. Quartz was inert at both temperatures, which indicates that the experimental procedure did not contaminate the samples. Overall, none of the minerals analyzed can be implicated in the natural jarosite clay low-temperature carbon dioxide peak, so combustion of organic matter remains the most likely source.

Variation in clay species does not appear to be an issue. Montmorillonite released water at both 600°C and 1000°C, and carbon dioxide appeared during the 1000°C step (Fig. 3). The carbon dioxide was again likely due to a carbonate impurity; a source by organic matter contamination is unlikely because of a lack of carbon dioxide detection at 600°C. Siderite decomposes at both 600°C and 1000°C to give carbon dioxide and a *m/z* 28 peak. The *m/z* 28 peak is interpreted as carbon monoxide rather than nitrogen due to the homogeneity of the sample and the decomposition products of siderite being well known (Gallagher and Warne, 1981). Although siderite is a potential source of carbon dioxide, the XRD and EA-IRMS analyses showed that carbonate was absent in the natural jarosite clay; thus siderite was not a plausible source of significant carbon dioxide in this sample. Gypsum was pyrolyzed, and as expected from previous work (West and Sutton, 1953) it only dehydrated, suggesting that this common sulfate is relatively stable at pyrolysis temperatures used for the detection of organic matter. In summary, our data from experiments studying the decomposition of other relevant mineral phases suggest that carbon dioxide released from the natural jarosite clay must primarily have originated from oxidation of organic matter by oxygen derived from jarosite decomposition.

### 3.6. Decomposition of ferric sulfates between 400°C and 1000°C

**FIG. 4.**
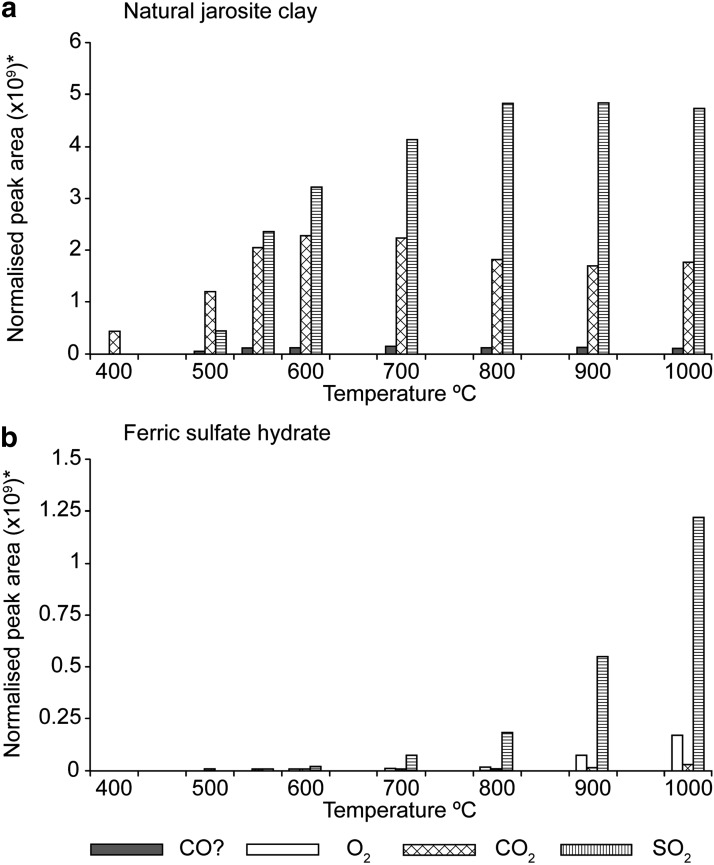
The production of sulfur dioxide, carbon dioxide, possible carbon monoxide, and oxygen during the thermal decomposition of samples of natural jarosite clay and a lab standard of ferric sulfate hydrate in individual heating experiments carried out between 400°C and 1000°C at 100°C increments. *The peak areas were normalized by sulfate mass (100% in ferric sulfate hydrate, 5% of sample mass in the natural jarosite clay—from XRD results). The *m/z* 28 peak is labeled as CO?, as it could be either carbon monoxide or nitrogen; we infer carbon monoxide as discussed in the text.

With the sources of the pyrolysis products from the decomposition of relevant mineral phases assessed, we performed further work to examine the relative response of pyrolysis products for the ferric sulfates with temperature (Fig. 4). The peak areas were normalized to the abundance of sulfate in the sample (100% for the ferric sulfate hydrate and 5% of sample mass for the natural jarosite clay).

The natural jarosite clay sample and synthetic ferric sulfate hydrate both began to decompose to produce sulfur dioxide at around the temperatures used for the flash pyrolysis of macromolecular organic matter (500–600°C). Yet the peak area for sulfur dioxide produced from jarosite decomposition was much greater than that seen in ferric sulfate. Sulfur dioxide contributions from other minerals in the sample are implausible sources because XRD indicated that there are no other sulfates present in the natural jarosite clay.

Oxygen was not detected during heating of the natural jarosite clay but was detectable in the ferric sulfate hydrate experiments at temperatures at and above 600°C (Fig. 4). A substantial response for carbon dioxide appears at low temperatures for the natural jarosite clay from 500°C, and a more limited response is evident at high temperatures from the ferric sulfate hydrate. The peak for the *m/z* 28 compound increases up to 550°C, and its response is relatively constant thereafter in the natural jarosite clay. The rapid increase of carbon dioxide at temperatures normally associated with the decomposition of organic matter suggests that the combustion of organic matter was aided by sulfate-sourced oxygen. The peak areas for sulfur dioxide, water, and the *m/z* 28 compound produced by the natural jarosite clay are very similar at 800°C and higher, indicating that the hydronium and ferric sulfate components in the potassium-hydronium jarosite were breaking down rapidly. The ferric sulfate hydrate peak areas were still increasing at 1000°C, indicating that full decomposition had not yet been achieved. Oxygen increased with sulfur dioxide in the ferric sulfate hydrate experiments. Jarosite appears to break down rapidly at temperatures commonly used in thermal decomposition studies of organic matter.

To better understand the production of sulfur dioxide and carbon dioxide during pyrolysis of the natural jarosite clay, the peak areas were quantified with reference to calibration curves (Table 2). Sulfur dioxide was calibrated by manual injections of known volumes of gas and carbon dioxide through the pyrolysis of different masses of sodium bicarbonate. If the entire carbon content (0.45%) of the natural jarosite clay was oxidized, then 1 mg would be expected to produce 0.0165 mg of carbon dioxide. The greatest mass of carbon dioxide produced per milligram of the natural jarosite clay was 0.0112 mg at 600°C, indicating that 68% of the carbon in the sample had been lost as carbon dioxide. Approximately a third of the organic carbon present must therefore be refractory and inaccessible by pyrolysis or combustion at temperatures ≤1000°C.

**Table 2.** Carbon Dioxide, Sulfur Dioxide, and Theoretical Oxygen Production per Milligram of Sample during Individual Pyrolysis Runs of the Natural Jarosite Clay

*Temperature (°C)*	*Mass SO_2_ (mg)*	*Theoretical mass of O_2_ release during sulfate decomposition*^a^*(mg)*	*Mass CO_2_ (mg)*	*C/O_2_ molar ratio*^b^
400	0	0	0.0021	—
500	0.0020	0.0010	0.0057	4.2
550	0.0104	0.0052	0.0099	1.4
600	0.0155	0.0078	0.0112	1.0
700	0.0190	0.0095	0.0110	0.8
800	0.0224	0.0112	0.0089	0.6
900	0.0228	0.0114	0.0085	0.5
1000	0.0217	0.0109	0.0087	0.6

^a^ During sulfate decomposition SO_2_ release is twice that of O_2_ (Holt and Engelkemeir, 1970).

^b^ Moles of carbon in CO_2_ detected divided by the moles of O_2_ released by sulfate decomposition. If the C/O_2_ ratio is at or below one, then there is theoretically enough O_2_ produced by sulfate decomposition to generate all the CO_2_ by oxidation of organic matter.

All the oxygen in the carbon dioxide mass produced at ∼600°C and above could be oxygen sourced from sulfate decomposition (Table 2). The lower-temperature carbon dioxide that is not sourced from oxidation must still be sourced from the thermal processing of organic carbon, as carbonates are not present in the sample. The decarboxylation of carboxylic acids (which were detected in the solvent extract) are likely to be responsible (Alencar *et al.,* 1983). Decarboxylation explains the mass of carbon dioxide detected at 400°C (Table 2), which appears before any sulfate decomposition occurs in the natural jarosite clay. Consequently, our data indicate that oxygen sourced from jarosite decomposition can oxidize a major proportion of indigenous organic carbon in a sample.

## 4. Discussion

### 4.1. Effects of ferric sulfates on past, present, and future Mars missions

It is evident from our data, therefore, that sulfate minerals represent a potential problem for the detection of organic matter on Mars by thermal extraction methods. Sulfates are abundant in martian rocks, regolith, and the well-mixed, globally spread dust. Here, it has been demonstrated that a well-mixed natural jarosite clay containing 5% jarosite can decompose at and above 500°C to release sulfur dioxide and, therefore, oxygen and in the process oxidize indigenous organic matter. Ferric sulfate hydrate also begins to decompose at similar temperatures to give sulfur dioxide. Gypsum and magnesium sulfate are unlikely to yield oxygen at the temperatures commonly employed for the thermal extraction of organic matter (Table 1). Much attention has been focused on the potential for perchlorate salts to disrupt thermal extraction experiments; it is the recommendation of the authors that ferric sulfates also be treated as a potential additional complication. Thermogravimetric studies suggest that aluminum sulfates may also disrupt organic detection experiments (Rudolph *et al.,* 2003).

Our newly acquired sulfate data can be used to reexamine past experiments on Mars. Viking lander thermal volatilization–gas chromatography–mass spectrometry (TV-GC-MS) data have been reinterpreted to suggest that the decomposition of minerals, specifically perchlorates, may have precluded the detection of organic matter (Navarro-González *et al.,* 2010). This reinterpretation was questioned by Biemann and Bada (2011), who stated that Viking data are not consistent with chlorination or oxidation of organic matter by perchlorates. Sulfates have not yet been directly considered as an additional confounding mineralogy during analysis on Mars. Inorganic chemical investigation on board the Viking Landers revealed high levels of sulfur (Clark *et al.,* 1977). However, sulfur-containing compounds were absent in data from pyrolysis experiments at temperatures of 500°C and below (Biemann *et al.,* 1977). Comparison of our data to those from the Viking experiments suggests that no potassium jarosite was present at the sample sites. Ferric sulfate may have been present, but pyrolysis temperatures would have been too low for substantial decomposition (Fig. 4). This comparison suggests that, even when confounding mineralogies exist, temperature windows can be identified within which pyrolysis studies can take place.

Jarosite was inferred as a possible contributor to a water release detected between 295°C and 735°C by TEGA at the Phoenix landing site (Smith *et al.,* 2009). However, no sulfur dioxide was detected in the heating range of 25–1000°C (Golden *et al.,* 2009). Our data indicate that jarosite can therefore be removed from the list of potential dehydrating minerals suggested by Smith *et al.* (2009). Gypsum was interpreted as a possible phase in the Phoenix landing site soil (Hecht *et al.,* 2009), an interpretation that is consistent with our data, which show that gypsum is unlikely to decompose to give sulfur dioxide below 1000°C.

Recently, pyrolysis of surface materials from the Rocknest eolian deposit in Gale Crater led to the simultaneous release of oxygen and chlorinated hydrocarbons, suggesting the influence of perchlorates on organic decomposition (Glavin *et al.,* 2013). Sulfur dioxide release was varied, suggesting heterogeneity in sulfur minerals, but a recognizable bimodal distribution existed with two peaks centered at ∼500°C to 550°C and ∼700°C to 750°C (Leshin *et al.,* 2013). Notably, the lower-temperature release of sulfur dioxide was accompanied by an increase in carbon dioxide. As the lower-temperature carbon dioxide peak is significantly larger than sulfur dioxide, it is primarily consistent with the thermal decomposition of siderite (Leshin *et al.,* 2013). However, it is possible that perchlorate decomposition-induced oxidation led to the degradation of organic matter at lower temperatures (*ca.* 350–450°C), while jarosite decomposition-induced oxidation at slightly higher temperature (*ca.* 450–550°C) exhausted any remaining organic matter, with the resulting carbon dioxide peak obscured by siderite decomposition (Leshin *et al.,* 2013). However, the carbon dioxide and carbon monoxide produced by siderite decomposition could be misinterpreted as evidence of the combustion of organic matter, so interpretation of siderite-containing samples is extremely challenging. Multiple minerals may be complicating the search for indigenous organic matter on Mars. MSL pyrolysis of mudstone samples at Yellowknife Bay also produced chlorinated hydrocarbons and a sulfur dioxide peak centered at 600°C. However, mudstones are indicative of neutral waters, rather than the low pH conditions needed for the formation of iron sulfates; thus sulfides are the more likely sulfur source at this locality (Ming *et al.,* 2014). Though sulfates are widespread on Mars, the types of sulfur species found at each locality seem to be highly variable.

The negative influence of sulfate minerals on organic detection is magnified when it is noted that organic matter and sulfates have been observed to colocate in terrestrial Mars analog environments. Sulfates are an indicator of the presence of past liquid water, which is a prerequisite for the creation of habitable environments that can support life and its biochemistry. Glycine has been shown to associate with terrestrial jarosite samples and influence their decomposition (Kotler *et al.,* 2009). It has been demonstrated that organic material can be preserved in ancient terrestrial sulfate minerals (Aubrey *et al.,* 2006). Sulfate-rich Mars analog sites, such as Río Tinto and Panoche Valley, were found to have severely attenuated organic responses in thermal volatilization experiments by Navarro-González *et al.* (2006), but the decomposition of sulfate ions to give oxygen was not one of the confounding mechanisms considered by the authors. It appears that sulfates on Mars may represent something of a mixed blessing, acting as an effective host for organic matter but also reflecting a potentially aggressive material that is difficult to analyze.

The possible colocation of organic matter with sulfate minerals on Mars makes avoiding sulfates an unsatisfactory mitigation step, and other options must be considered. Only certain sulfates decompose to produce sulfur dioxide and then oxygen at the temperatures used for the detection of organic matter. A preliminary mineralogical analysis allows the risk of sulfate interference to be assessed. Such a method is used by MSL, where SAM results can be compared with data from the CheMin instrument (Glavin *et al.,* 2013). If jarosite is detected, it would be a strong contender for the source of sulfur dioxide, water, and potentially oxygen and high-temperature carbon dioxide peaks (the latter being derived from the oxidation of organic matter) seen in thermal extraction experiments. In addition, the carbon dioxide peaks resulting from perchlorate and/or sulfate oxidation on Mars can be interpreted to indicate the masses of organic compounds being oxidized and potentially differentiate contamination from indigenous organic compounds (Sephton *et al.,* 2014).

## 5. Conclusions

Sulfate minerals are commonly found on Mars. Iron sulfate species such as jarosite and ferric sulfate experience breakdown of their sulfate structures and therefore release oxygen around pyrolysis temperatures used to thermally extract organic matter. Sulfate decomposition can lead to the introduction of oxygen and/or sulfuric acid to pyrolysis chambers. Martian ferric sulfates therefore represent a significant complication to organic detection through heat-based methods. A large peak of carbon dioxide and a minor peak likely representing carbon monoxide were detected during pyrolysis of a natural jarosite clay sample, suggesting that the organic content is being combusted by oxygen released by jarosite decomposition. The prevalence of sulfates in martian rocks, regolith, and the globally mixed dust means that they are difficult to avoid. In addition, terrestrial sulfates have been shown to associate with organic compounds, so avoiding these minerals on Mars during organic detection experiments would be unwise. Previous authors have recognized that the decomposition of perchlorates on Mars may degrade organic matter. Sulfates may also be contributing to the oxidation of organic matter during thermal analyses, thereby complicating interpretations. However, the relatively high breakdown temperatures for sulfates mean that a combination of preliminary mineralogical analyses and suitably selected thermal extraction temperatures may minimize or remove their negative influence.

## Abbreviations Used

CheMin: Chemistry and Mineralogy
EA-IRMS: elemental analyzer–isotope ratio mass spectrometry
GC-MS: gas chromatography–mass spectrometry
MSL: Mars Science Laboratory
*m/z*: mass-to-charge ratio
Py-GC-MS: pyrolysis–gas chromatography–mass spectrometry
SAM: Sample Analysis at Mars
TEGA: Thermal Evolved Gas Analyzer
XRD: X-ray diffraction
